# General metabolism of *Laribacter hongkongensis*: a genome-wide analysis

**DOI:** 10.1186/2045-3701-1-16

**Published:** 2011-04-19

**Authors:** Shirly O Curreem, Jade L Teng, Herman Tse, Kwok-Yung Yuen, Susanna K Lau, Patrick C Woo

**Affiliations:** 1 Department of Microbiology, The University of Hong Kong, Hong Kong; 2 State Key Laboratory of Emerging Infectious Diseases, Hong Kong; 3 Research Centre of Infection and Immunology, The University of Hong Kong, Hong Kong; 4 Carol Yu Centre of Infection, The University of Hong Kong, Hong Kong

## Abstract

**Background:**

*Laribacter hongkongensis *is associated with community-acquired gastroenteritis and traveler's diarrhea. In this study, we performed an in-depth annotation of the genes and pathways of the general metabolism of *L. hongkongensis *and correlated them with its phenotypic characteristics.

**Results:**

The *L. hongkongensis *genome possesses the pentose phosphate and gluconeogenesis pathways and tricarboxylic acid and glyoxylate cycles, but incomplete Embden-Meyerhof-Parnas and Entner-Doudoroff pathways, in agreement with its asaccharolytic phenotype. It contains enzymes for biosynthesis and β-oxidation of saturated fatty acids, biosynthesis of all 20 universal amino acids and selenocysteine, the latter not observed in *Neisseria gonorrhoeae*, *Neisseria meningitidis *and *Chromobacterium violaceum*. The genome contains a variety of dehydrogenases, enabling it to utilize different substrates as electron donors. It encodes three terminal cytochrome oxidases for respiration using oxygen as the electron acceptor under aerobic and microaerophilic conditions and four reductases for respiration with alternative electron acceptors under anaerobic conditions. The presence of complete tetrathionate reductase operon may confer survival advantage in mammalian host in association with diarrhea. The genome contains CDSs for incorporating sulfur and nitrogen by sulfate assimilation, ammonia assimilation and nitrate reduction. The existence of both glutamate dehydrogenase and glutamine synthetase/glutamate synthase pathways suggests an importance of ammonia metabolism in the living environments that it may encounter.

**Conclusions:**

The *L. hongkongensis *genome possesses a variety of genes and pathways for carbohydrate, amino acid and lipid metabolism, respiratory chain and sulfur and nitrogen metabolism. These allow the bacterium to utilize various substrates for energy production and survive in different environmental niches.

## Background

In 2001, *Laribacter hongkongensis*, a novel genus and species that belongs to the *Neisseriaceae *family of β-subclass of the Proteobacteria, was discovered from the blood and empyema pus of a patient with underlying alcoholic cirrhosis [1]. Subsequently, it was observed that *L. hongkongensis *was associated with freshwater fish borne community-acquired gastroenteritis and traveler's diarrhea in human [2-6]. In addition to its capability of living under both aerobic and anaerobic conditions and in the intestines of human, a variety of freshwater fish and frogs, it can also survive and replicate as a free living bacterium in water obtained from drinking water reservoirs [6-10]. Despite its capability of survival in diverse environmental conditions, it does not metabolize any sugar tested [1,3,4,11].

In this article, we present an overview of the general metabolism of *L. hongkongensis *based on the information obtained from its genome analysis. The metabolic pathways of *L. hongkongensis *were also compared to those of *Neisseria gonorrhoeae*, *Neisseria meningitidis*, *Chromobacterium violaceum*, *Escherichia coli *and *Campylobacter jejuni. N. gonorrhoeae*, *N. meningitidis *and *C. violaceum *are the other three bacterial species in the *Neisseriaceae *family of β-Proteobacteria with complete genome sequences available [12-15]. *N. gonorrhoeae *and *N. meningitidis *are strict aerobes that have stringent growth requirements and humans are their only known reservoir and host [16]. Conversely, *C. violaceum *is facultative anaerobic, highly versatile in its metabolism, and can be found abundantly in multiple ecosystems, including water and soil; and in tropical and subtropical regions [17]. *E. coli *is the prototype Gram-negative bacterium with its metabolic pathways dissected in the greatest detail. *C. jejuni *is another Gram-negative, S-shaped, motile, asaccharolytic bacillus associated with gastroenteritis [18].

## Results and discussion

**Figure 1:**
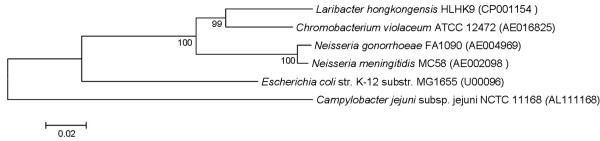
**Phylogenetic relationship of 16S rRNA among *L. hongkongensis *HLHK9*, N. meningitidis *MC58*, N. gonorrhoeae *FA 1090*, C. violaceum *ATCC 12472*, E. coli *K12 MG1655 and *C. jejuni *NCTC 11168**. The tree was inferred from 16S rRNA data by the neighbor-joining method. Bootstrap values were calculated from 1,000 trees. The scale bar indicates the estimated number of substitutions per 50 bases. Names and accession numbers are given as cited in the GenBank database.

The general metabolism discussed in this context mainly focuses on coding sequences (CDSs) that were classified into Cluster of Orthologous Groups (COG) functional categories of group C (energy production and conversion), G (carbohydrate transport and metabolism), E (amino acid transport and metabolism), I (lipid transport and metabolism) and P (inorganic ion transport and metabolism). Overall, the number of CDSs that were classified into these COGs was 191, 92, 253, 85 and 155 respectively in *L. hongkongensis *genome. These numbers are higher than those in *N. gonorrhoeae *and *N. meningitidis *genomes (113, 61, 146, 44 and 96 CDSs respectively and 120, 67, 156, 52 and 96 CDSs respectively) but lower than those in *C. violaceum *genome (208, 203, 423, 139 and 226 CDSs respectively). This large number of genes in *C. violaceum *genome is in line with its ability to survive in a wide range of environments, whereas the relatively smaller number of genes in the genomes of *N. gonorrhoeae *and *N. meningitidis *reflects their fastidious growth requirements and limited host ranges. Phylogenetic relationship of *L. hongkongensis *and other bacteria that are included for comparative analysis in this study based on 16S rRNA gene shows that *L. hongkongensis *is most similar to *C. violaceum *within the *Neisseriaceae *family with complete genome sequences available (Figure 1). This taxonomic closeness is also reflected in its gene contents, in which *L. hongkongensis *shares the highest percentage of CDSs with *C. violaceum *(64.1%) compared with other bacteria (Table 1).

**Table 1:** Gene contents in *L. hongkongensis*, *N. meningitidis*, *N. gonorrhoeae*, *C. violaceum*, *E. coli* and *C. jejuni*

	*L. hongkongensis *HLHK9	*C. violaceum *ATCC 12472	*N. gonorrhoeae* FA 1090	*N. meningitidis* MC58	*E. coli *K12 MG1655	*C. jejuni* NCTC 11168
Total number of CDSs	3235	4407	2002	2143	4321	1647
With homologues in L. hongkongensis	-	64.1% (2075/3235)	39.6% (1282/3235)	40.0% (1295/3235)	50.4% (1629/3235)	24.6% (795/3235)
RNA gene						
rRNA genes						
5S rRNA	7	9	4	4	8	3
16S rRNA	7	8	4	4	7	3
23S rRNA	7	8	4	4	7	3
tRNA genes	78	98	55	59	89	44

**Figure 2:**
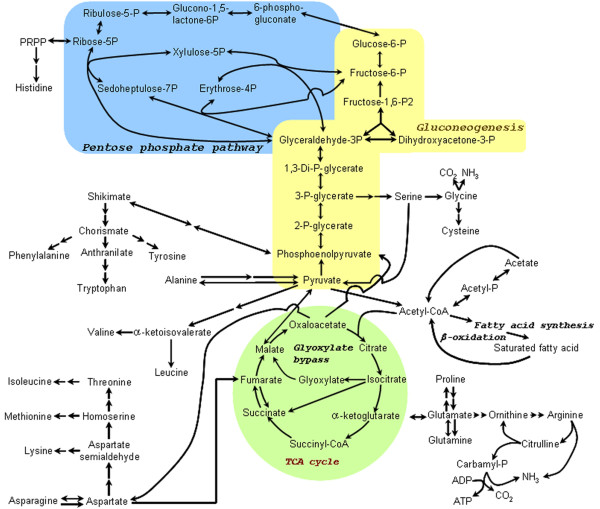
**Central metabolic pathways of *L. hongkongensis *HLHK9 deduced from its genome sequence**. The intermediary metabolic steps of tricarboxylic acid (TCA) cycle, gluconeogensis, glyoxylate cycle, pentose phosphate pathway, fatty acid and amino acid metabolism are shown. The function predictions were based on sequence similarity to proteins with experimentally verified functions. Individual pathways are shaded as indicated: yellow for gluconeogenesis, green for TCA cycle, and blue for pentose phosphate pathway. Arrows indicate the flow of intermediates.

Figure 2 illustrates the deduced central metabolism of *L. hongkongensis *from the genomic data. The reconstructed metabolic pathways reconciled the results of previous physiological and biochemical studies in light of the metabolic capacity of this strain and showed remarkable ability to adapt to diverse environment. Comparison with other *Neisseriaceae *bacteria, prototype *E. coli *and *C. jejuni *brings to light the unique capabilities of *L. hongkongensis*. Utilization of compounds as carbon and energy sources was more restricted than in *C. violaceum*, but more extended than *N. meningitidis *and *N. gonorrhoeae*.

### Carbohydrate metabolism

In most bacteria, glucose is metabolized through Embden-Meyerhof-Parnas (EMP) pathway, a pathway that is also highly conserved in eukaryotes and archaea. Apart from EMP pathway, some prokaryotes adopt Entner-Doudoroff (ED) pathway for sugar metabolism by priming of 6-carbon sugars and subsequent cleavage into two 3-carbon intermediates by aldolase enzymes [19]. While some bacteria, such as *E. coli *and *Pseudomonas aeruginosa*, have genes for both EMP and ED pathways, others contain only either one of them, or each pathway is dedicated for the metabolism of specific 6-carbon sugar [19]. In *L. hongkongensis*, both pathways were incomplete with regards to the predicted genes present in the genome (Figure 2). The absence of hexokinase, 6-phosphofructokinase and pyruvate kinase, which are key enzymes of EMP pathway, suggested that the bacterium cannot catabolize glucose through EMP pathway, in agreement with its asaccharolytic phenotype. Similar observations are found in other asaccharolytic bacteria such as *C. jejuni*, *Bordetella pertussis*, *Bordetella parapertussis *and *Bordetella bronchiseptica*, in which the genes encoding glucokinase and 6-phosphofructokinase are also absent from their genomes [15,20]. For *N. gonorrhoeae *and *N. meningitidis*, although they both metabolize a variety of sugars, their genomes do not contain 6-phosphofructokinase gene. Instead, they use ED pathway for metabolizing the 6-carbon sugars [12,21]. In the genome of *L. hongkongensis*, the gene that encodes one (2-keto-3-deoxy-6-phosphogluconate aldolase), but not the other (6-phosphogluconate dehydratase), of the two key enzymes of ED pathway is present, suggesting an incomplete pathway exists for metabolizing 6-carbon sugars through this pathway. With the inability to utilize exogenous carbohydrates, it is thus expected that gluconeogenesis would be essential to generate 6-carbon intermediates required for other biosynthetic pathways in *L. hongkongensis*, and the presence of complete gluconeogenic genes is coherent with this prediction (Figure 2).

In line with the asaccharolytic nature of *L. hongkongensis *and *C. jejuni*, both genomes do not contain genes that encode a complete phosphoenolpyruvate-dependent, sugar transporting phosphotransferase system (PTS), one of the major carbohydrate transport systems in bacteria. The genome of *L. hongkongensis *contains genes that encode enzyme I, phosphocarrier protein HPr, HPr kinase/phosphorylase HPrK, enzyme IIA^Ntr ^and enzyme IIA^fru^, whereas none of the genes that encode any of the components of PTS is present in the genome of *C. jejuni *[15]. Similar to *L. hongkongensis*, *N. meningitidis *and *N. gonorrhoeae *also contain cytoplasmic PTS protein homologues including EI, NPr (a variant of HPr) and HPrK together with incomplete enzyme II homologues although they were both saccharolytic. The presence of other sugar permeases in their genome suggests that sugars are probably transported through other pathways. In the genomes of *C. violaceum *and *E. coli*, genes encoding complete PTS protein homologues (enzyme I and HPr) and multiple types of enzyme II permeases are present, compatible with their ability to use a large number of sugars [13,14]. In general, enzyme I and HPr are energy coupling proteins of the system while complete enzyme II complexes, PTS permeases, consist of IIA, IIB and IIC domains (as well as IID domain in the family of mannose). Instead of carbohydrate transport, some enzymes may be involved in regulation [22]. The presence of enzyme IIA^Ntr ^and enzyme IIA^fru ^in *L. hongkongensis *suggested that they are probably needed for the regulation of other biochemical pathways rather than sugar transport.

Genes encoding enzymes of both non-oxidative and oxidative branches of pentose phosphate pathway (PPP) and TCA cycle can be found in the genome of *L. hongkongensis*, *C. violaceum *and *E. coli*, whereas *C. jejuni *lacks the oxidative branch of PPP [11-15]. Although genes that encode enzymes for PPP and most of TCA cycle are present in the genomes of *N. meningitidis *and *N. gonorrhoeae*, the gene for malate dehydrogenase is absent from both genomes. The presence of flavin adenine dinucleotide (FAD)-dependent malate:quinine oxidoreductase may substitute for the reaction catalyzed by malate dehydrogenase by oxidizing malate to oxaloacetate, yet experimental analysis awaits to confirm such prediction [21]. When carbon sources are metabolized to acetyl-coenzyme A (acetyl-CoA) instead of pyruvate or phosphoenolpyruvate, TCA cycle intermediates cannot be replenished through anaplerotic reactions. A complete pathway for glyoxylate cycle found in *L. hongkongensis *provides a simple and efficient strategy for it to convert acetyl-CoA into anaplerotic and gluconeogenic compounds and enables it to grow with C-2 compounds as the sole carbon source. With glyoxylate pathway, gluconeogenesis, non-oxidative PPP and anaplerotic reactions, *L. hongkongensis *will therefore be able to generate different intermediates required for biosynthetic metabolism. The presence of critical anaplerotic enzymes, including phosphoenolpyruvate synthase and phosphoenolpyruvate carboxylase, which convert pyruvate and oxaloacetate respectively to phosphoenolpyruvate, together with malate oxidoreductase in *L. hongkongensis *reflects its potential to grow on carbon sources such as malate (Table 2). Malate, together with succinate and fumarate, are C_4_-dicarboxylates. They are intermediates in TCA cycle that can be utilized by bacteria such as *E. coli*, *B. subtilus *and many *Rhizobium *spp. as nonfermentable carbon and/or energy sources under aerobic or anaerobic conditions [23]. A number of C_4_-dicarboxylates can be found in nature such as fermentation products (e.g. succinate) and complexing agents (e.g. oxalate and malate) [24]. The presence of several C_4_-dicarboxylates transporters reinforces the prediction of using C_4_-dicarboxylates as carbon sources in *L. hongkongensis*. This is in line with our experiments on minimal medium for *L. hongkongensis *which showed that L-malate can be used as sole carbon source [11].

**Table 2:** CDSs related to anaplerotic reactions in *L. hongkongensis*, *N. meningitidis*, *N. gonorrhoeae*, *C. violaceum*, *E. coli* and *C. jejuni*

Enzyme	Gene	*L. hongkongensis *HLHK9	*C. violaceum *ATCC 12472	*N. gonorrhoeae* FA 1090	*N. meningitidis* MC58	*E. coli *K12 MG1655	*C. jejuni* NCTC 11168
Phosphoenolpyruvate carboxylase	*ppc*	+	+	+	+	+	-
Phosphoenolpyruvate synthase	*pps*	+	+	+	+	+	-
Phosphoenolpyruvate carboxykinase	*pck*	-	-	-	-	+	+
Malate oxidoreductase (EC 1.1.1.38)	*maeA*	-	-	+	+	+	+
Malate oxidoreductase (EC 1.1.1.40)	*maeB*	+	+	-	-	+	-

### Amino acid metabolism

Similar to *E. coli *and *C. jejuni*, but not *N. gonorrhoeae*, *N. meningitidis *and *C. violaceum*, the genome of *L. hongkongensis *contains enzymes for biosynthesis of all 20 universally found amino acids and also selenocysteine. Selenocysteine (Sec) is an amino acid present in many species in three domains of life [25,26]. It is encoded by opal codon (UGA), of which selenoprotein mRNA carries a selenocysteine insertion sequence element immediately downstream to the selenocysteine-encoding UGA codon [27]. Biosynthesis and incorporation of selenocysteine requires four genes, including *selA *(encoding Sec synthase), *selB *(encoding Sec-specific elongation factor), *selC *(encoding tRNA^Sec^) and *selD *(encoding selenophosphate synthetase), whereas *ybbB *(encoding tRNA 2-selenouridine synthase) is needed for utilization. All the genes required for biosynthesis and incorporation of selenocysteine are present in the genomes of about 20% of bacteria with complete genome sequence available, with a majority in Proteobacteria and Firmicutes [28]. While *L. hongkongensis *is predicted to synthesize selenocysteine, such ability is not found in *C. violaceum*, *N. gonorrhoeae *and *N. meningitidis *which also belong to β-Proteobacteria. Although the reason for the selective presence of selenocysteine in some organisms remains unknown, it is usually present in the active sites of proteins with redox functions [27]. From the genomic data, it is predicted that only the most commonly found selenoprotein, alpha subunit of formate dehydrogenase encoded by *fdoG*, is present in *L. hongkongensis *using a selenoprotein prediction software bSECISearch [28,29].

Similar to *C. jejuni*, the genome of *L. hongkongensis *contains a number of proteases, peptidases and transporters for degradation and transport of proteins or peptides into oligopeptides or amino acids. The amino acids can then be degraded intracellularly by various enzymes (Table 3). These amino acids can be used as carbon and nitrogen source for the bacterium. The products of the reactions can enter central metabolic pathways including TCA cycle and gluconeogenesis pathway, and can be used for anabolic and catabolic purposes (Figure 2 and Table 3).

**Table 3:** Amino acid catabolism of *L. hongkongensis *HLHK9 deduced from its genome sequence

Amino acid	Pathway/enzyme(s) involved	Gene number	Intermediates/products formed	Pathways that intermediates enter
Serine	L-serine dehydratase	LHK_02265	Pyruvate, ammonia	TCA cycle or gluconeogenesis
Aspartate	Argininosuccinate synthase	LHK_02172	Fumarate, oxaloacetate, ammonia, ATP	TCA cycle
	Argininosuccinate lyase	LHK_03122		
	L-aspartate oxidase	LHK_00001		
	Aspartate aminotransferase	LHK_01340		
Glutamine	Glutamine synthetase	LHK_01876	α-ketoglutarate, ammonia	TCA cycle
	Glutamate dehydrogenase	LHK_01886		
Glycine	Glycine cleavage system P-protein	LHK_02722	Ammonia, CO_2_, NADH	Ammonia assimilation
	Glycine cleavage system H protein	LHK_02723		
	Glycine cleavage system T protein	LHK_02724		
Alanine	Alanine dehydrogenase	LHK_02210	Pyruvate	TCA cycle or gluconeogenesis
	Alanine racemase	LHK_00350		
	D-amino acid dehydrogenase	LHK_00934		
Glutamate	Glutamate dehydrogenase	LHK_01886	α-ketoglutarate, ammonia	TCA cycle
Arginine	Arginine deiminase pathway		Ammonia, ATP	Ammonia assimilation
	Arginine deiminase	LHK_02729, LHK_02734		
	Ornithine carbamoyltransferase	LHK_02728, LHK_02733		
	Carbamate kinase	LHK_02727, LHK_02732		
	Arginine decarboxylase pathway		Putrescine	TCA cycle
	Arginine decarboxylase	LHK_01034		
	Agmatinase	LHK_01140		
Proline	Proline dehydrogenase	LHK_01861	α-ketoglutarate, ammonia	TCA cycle
	1-pyrroline carboxylate dehydrogenase	LHK_01861		

As predicted from the genomic sequence, several genes involved in the biosynthesis (amino-acid acetyltransferase gene *argA*: LHK_02338, LHK_02366*; *acetylglutamate kinase gene *argB: *LHK_02337, LHK_02829) and catabolism (whole operon of arginine deiminase *arcB*/*A*/*C*/*D*: LHK_02727-LHK_02734) of arginine are duplicated in *L. hongkongensis*, suggesting the importance of arginine metabolism in the bacterium. Previously, we have shown that the two isoenzymes of *N*-acetyl-L-glutamate kinase (NAGK) encoded by duplicated copies of *argB*, NAGK-20 and NAGK-37, which catalyze the key reaction in the 8-step arginine biosynthesis, gave differential expression pattern in a comparative proteomic study of *L. hongkongensis *growing at 37°C (human body temperature) and 20°C (freshwater habitat temperature) [11]. With NAGK-20 showing a higher expression at 20°C and NAKG-37 showing a higher expression at 37°C, kinetic analysis revealed that NAGK-20 also had a lower optimal temperature for enzymatic activities and was inhibited by arginine whereas NAGK-37 had a higher enzymatic activity with a higher optimal temperature and was insensitive to arginine inhibition [11]. These observations suggest the two isoenzymes are involved in temperature adaptation. Further investigation into the functions of duplicated genes in the metabolic pathway of arginine should yield fruitful insights into the lifestyle of *L. hongkongensis*.

### Lipid metabolism

Fatty acids are synthesized via repeated cycles of condensation, dehydration and reduction of carbon-carbon bonds. While the majority of bacterial membranes are composed of saturated fatty acids, presence of unsaturated fatty acids (UFAs) can increase the fluidity of the membrane [30,31]. Similar to the genomes of the other five bacteria, the genome of *L. hongkongensis *contains all the enzymes for biosynthesis of saturated fatty acids (Table 4). As for UFA biosynthesis, there are two known sets of enzymes that operate by different mechanisms: aerobic route and anaerobic route [32]. In the aerobic route, UFAs are formed by the oxidation of saturated fatty acids catalyzed by acyl-ACP oxidase (fatty acid desaturase). In the anaerobic route, *trans *double bond is introduced to the acyl chain of ß-hydroxy-decanoyl-ACP and isomerized to *cis*-3-decenoyl-ACP by bifunctional 3-hydroxydecanoyl-ACP dehydratase/isomerase (FabA) (encoded by *fabA*), followed by 3-ketoacyl-ACP synthase I (FabB) (encoded by *fabB*) which catalyzes the elongation of *cis*-3-decenoyl-ACP to form UFAs. This pathway is well-studied in *E. coli *[32,33]. The presence of either mechanism is sufficient for the biosynthesis of UFA. Unlike *C. violaceum*, which has the genes for the aerobic UFA biosynthetic pathway, the genome of *L. hongkongensis *does not contain the gene that encodes desaturase. As for the anaerobic route, *fabA*-*fabB *is generally restricted to genomes of α- and γ-Proteobacteria. Therefore, it is not surprising that the genomes of *L. hongkongensis*, *N. gonorrhoeae*, *N. meningitidis *and *C. violaceum *and that of *C. jejuni *(ε-Proteobacteria) do not contain *fabA*-*fabB*. Alternative pathways for anaerobic UFA biosynthesis in the absence of *fadA *and *fadB *have been reported in other bacteria such as *Streptococcus pneumoniae *and *Enterococcus faecalis*, in which a *trans*-2 to *cis*-3-decenoyl-ACP isomerase (FabM) can introduce a *cis *double bond into the growing acyl chain in the former and the presence of homologues of FabZ and FabF that can function as FabA and FabB in the latter [34,35]. Other options such as dual functions of FabF involved in saturated fatty acid biosynthesis as FabF and UFA biosynthesis as FabB has also been reported in *Clostridium acetobutylicium *and *Lactococcus lactis *[36,37]. While *L. hongkongensis *contains neither of the alternative *fabA*, *fabB *and *fabM *genes in its genome, whether its FabF has dual function is yet to be determined. This phenomenon has also been observed in the genomes of *N. gonorrhoeae *and *N. meningitidis*. It has been suggested that there may be uncharacterized enzymes and pathways for unsaturated fatty acid biosynthesis in these bacteria [38]. Analysis of membrane phospholipids in *L. hongkongensis *will reveal the types of unsaturated fatty acids that it possesses and help to delineate the possible biosynthetic pathway.

**Table 4:** Comparison of metabolic pathways for fatty acid metabolism deduced from the genomes of *L. hongkongensis*, *N. meningitidis*, *N. gonorrhoeae*, *C. violaceum*, *E. coli* and *C. jejuni*

Pathway	*L. hongkongensis *HLHK9	*C. violaceum *ATCC 12472	*N. gonorrhoeae* FA 1090	*N. meningitidis *MC58	*E. coli *K12 MG1655	*C. jejuni *NCTC 11168
Fatty acid biosynthesis						
Saturated fatty acid	***+***	***+***	***+***	***+***	***+***	***+***
Unsaturated fatty acid	-	+	-	-	+	-
Cyclopropane fatty acid	+	+	-	-	+	+
Fatty acid catabolism						
Saturated fatty acid	+	+	-	-	+	-
Unsaturated fatty acid	-	+	-	-	+	-

In addition, the presence of two homologues of cyclopropane fatty-acyl-phospholipid synthases (LHK_01324 and LHK_03103) (CFA synthase) suggested the possibility for *L. hongkongensis *to synthesize cyclopropane fatty acid. Cyclopropane fatty acids are found in the bacterial membrane and are believed to be involved in acid resistance in *E. coli *and in association with virulence and persistence of *Mycobacterium tuberculosis *in host [39,40].

For the catabolism of fatty acids, genes that encode complete set of enzymes for β-oxidation pathway of saturated fatty acids are present in the genome of *L. hongkongensis *(Table 4). This suggests that *L. hongkongensis *may utilize these fatty acids as an energy source. The product of β-oxidation of saturated fatty acids, acetyl-CoA, will enter TCA cycle. On the other hand, similar to *N. gonorrhoeae*, *N. meningitidis *and *C. jejuni*, the genome of *L. hongkongensis *does not contain any of the two genes that encode enzymes for catabolism of unsaturated fatty acids (Table 4).

### Respiratory chain

The constituents of the respiratory chain of *L. hongkongensis *dissected under aerobic and anaerobic growth conditions revealed the metabolic potential to obtain energy from various sources. The primary function of respiratory chain of bacteria is to produce ATP through the transport of electrons from various electron donors, usually intermediates of metabolic pathways linked by quinones or ubiquinones, to various electron acceptors. The proton gradient generated between the cytoplasm and periplasm is then dissipated for ATP synthesis by F_1_F_0 _ATP synthase, which is predicted to be present in the genomes of *L. hongkongensis *and the other 5 species compared in this context [32]. The genome of *L. hongkongensis *contains a plethora of dehydrogenases, including NADH dehydrogenase I, Rnf type electron transport complex, succinate dehydrogenase, formate dehydrogenase, proline dehydrogenase, electron-transferring flavoprotein dehydrogenase, and D-amino acid dehydrogenase (Table 5). A variety of substrates as electron donors, such as NADH, succinate, formate, proline, acyl-CoA and D-amino acids appears to be utilized. The Rnf type electron transport complex was found in the genomes of *L. hongkongensis *and *E. coli*, but not those of *N. gonorrhoeae*, *N. meningitidis*, *C. violaceum *and *C. jejuni*. The complex was first discovered in *Rhodobacter capsulatus *in 1993 [41]. Although its role is not fully established, it was proposed to be an alternative enzyme to NADH dehydrogenase for utilizing NADH as the electron donor and had been found in many bacteria [42-44].

**Table 5:** Comparison of components of the respiratory chains deduced from the genomes of *L. hongkongensis*, *N. meningitidis*, *N. gonorrhoeae*, *C. violaceum*, *E. coli* and *C. jejuni*

Pathway/enzymes	*L. hongkongensis *HLHK9	*C. violaceum *ATCC 12472	*N. gonorrhoeae* FA 1090	*N. meningitidis *MC58	*E. coli *K12 MG1655	*C. jejuni *NCTC 11168
Electron donors						
NADH dehydrogenase^a^	I	I and II	I and II	I and II	I and II	I^b^
Succinate dehydrogenase	+	+	+	+	+	+
Rnf-type electron transport complex	+	-	-	-	+	-
Formate dehydrogenase	+	+	-	-	+	+
Proline dehydrogenase	+	+	+	+	+	+
Electron-transferring flavoprotein dehydrogenase	+	+	+	+	+	-
D-amino acid dehydrogenase	+	+	+	+	+	-
Electron acceptors for aerobic respiration						
Cytochrome oxidase (*aa_3_*type)	+	+	-	-	+	-
Cytochrome oxidase (*cbb_3_*type)	+	+	+	+	-	+
Cytochrome oxidase (*bd *type)	+	+	-	-	+	+
Electron acceptors for anaerobic respiration						
Periplasmic nitrate reductase Nap type	+	*napA *only^c^	-	-	+	+
Nitrate reductase Nar type	-	+	-	-	+	-
Fumarate reductase	+	+	+	+	+	+
DMSO reductase	+	-	-	-	+	-
Tetrathionate reductase	+	-	-	-	-	-
F_1_F_0_ATP synthase	+	+	+	+	+	+

^a^Two types of NADH dehydrogenases (NDH) can function in the respiratory complex I in bacteria. Type I NDH is a proton pumping NADH:ubiquinone oxidoreductase that transfer electron from NADH to ubiquinone for generation of electrochemical proton gradient [75]. Type II NDH is a monomeric enzyme in which NADH oxidation is not coupled to proton pumping [76].

^b^The gene cluster that encodes NADH dehydrogenase I in most bacteria are consisted of 14 different genes. *C. jejuni *contains 12 of the 14 genes in which *nuoE *and *nuoF *are absent [15].

^c^The basic gene components for periplasmic Nap type nitrate reductase to be regarded as functional are *napABCD *[68]. *C. violaceum *contains only *napA*.

**Figure 3:**
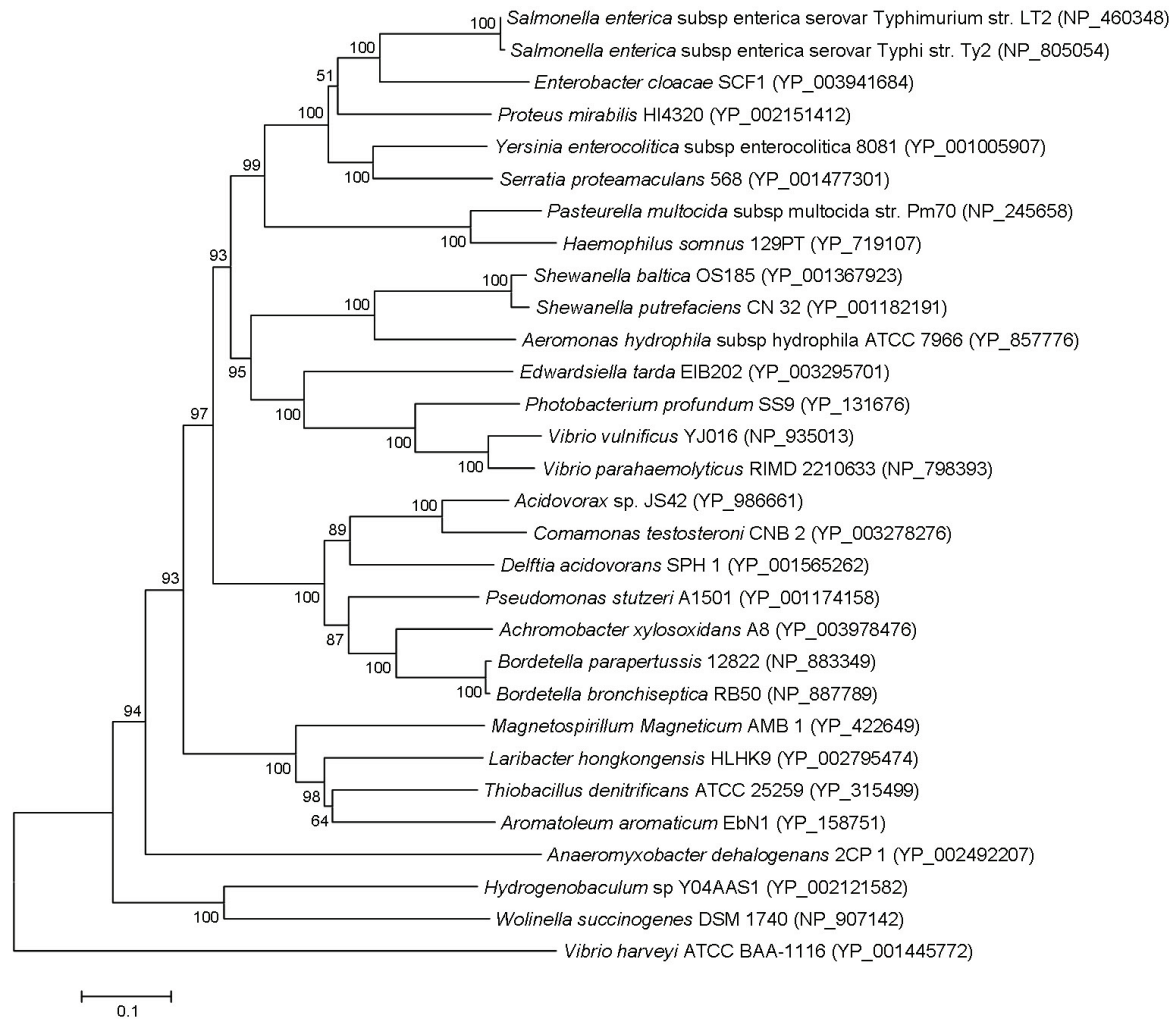
**Phylogenetic analysis of *ttrA *encoded protein in tetrathionate reductase gene cluster in *L. hongkongensis *HLHK9**. Phylogenetic tree showing the relationship of *ttrA* encoded proteins of *L. hongkongensis* HLHK9 to other species with complete *ttr* gene cluster constructed by neighbor-joining method. Bootstrap values were calculated from 1000 trees. The scale bar indicates the estimated number of substitutions per 10 amino acids. All names and accession numbers are given as cited in the GenBank database.

**Figure 4:**
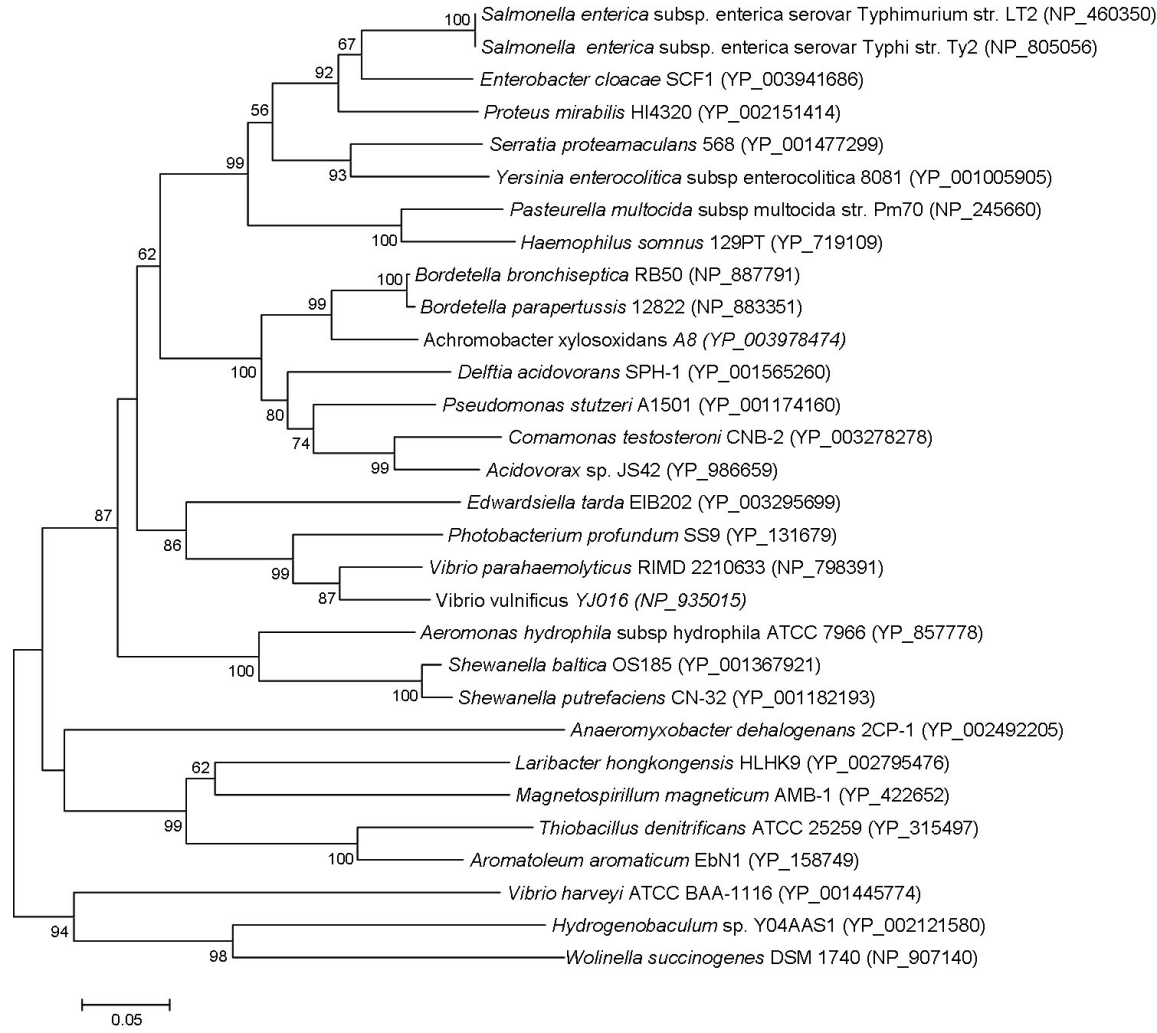
**Phylogenetic analysis of *ttrB *encoded protein in tetrathionate reductase gene cluster in *L. hongkongensis* HLHK9**. Phylogenetic tree showing the relationship of *ttrB* encoded proteins of *L. hongkongensis* HLHK9 to other species with complete *ttr* gene cluster constructed by neighbor-joining method. Bootstrap values were calculated from 1000 trees. The scale bar indicates the estimated number of substitutions per 20 amino acids. All names and accession numbers are given as cited in the GenBank database.

**Figure 5:**
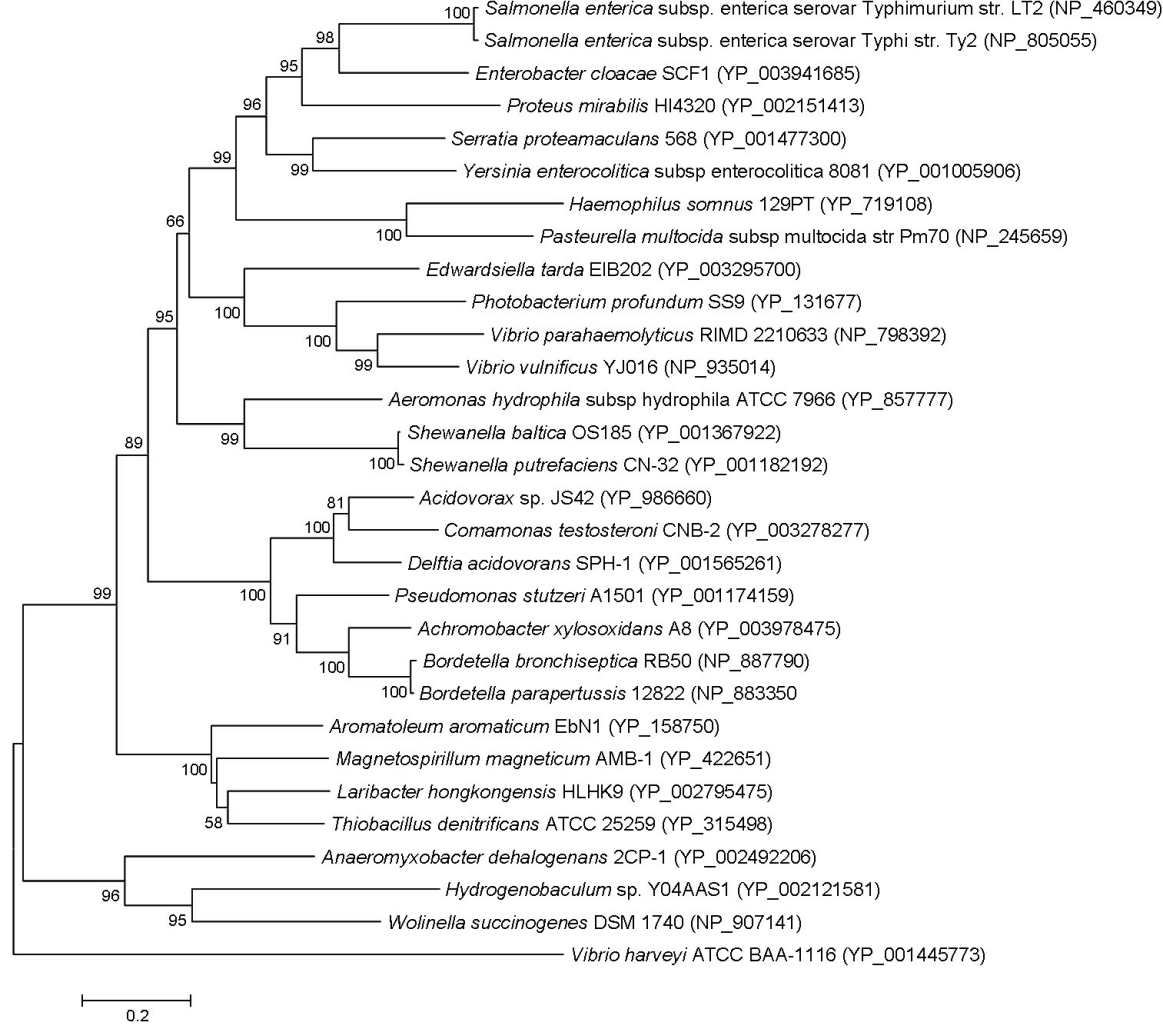
**Phylogenetic analysis of *ttrC *encoded protein in tetrathionate reductase gene cluster in *L. hongkongensis* HLHK9**. Phylogenetic tree showing the relationship of *ttrC* encoded proteins of *L. hongkongensis* HLHK9 to other species with complete *ttr* gene cluster constructed by neighbor-joining method. Bootstrap values were calculated from 1000 trees. The scale bar indicates the estimated number of substitutions per 5 amino acids. All names and accession numbers are given as cited in the GenBank database.

As for the final step of the respiratory chain, *L. hongkongensis *genome encodes three terminal cytochrome oxidases, namely type *aa_3 _*oxidase (a haem-copper oxidase), type *cbb_3 _*oxidase (another haem-copper oxidase) and type *bd *oxidase (a quinol oxidase). These three cytochrome oxidases are responsible for carrying out respiration using oxygen as the electron acceptor under aerobic conditions (type *aa_3 _*oxidase) and conditions with reduced oxygen tension (type *cbb_3 _*and type *bd *oxidases) (Table 5). Among the three cytochrome oxidases, type *cbb_3_*oxidase is the most ancient one and is present in almost all Proteobacteria except anaerobic δ-Proteobacteria [45]. It is characterized by the high affinity for oxygen which is needed in microaerobic environment. In addition to cytochrome oxidases, *L. hongkongensis *genome also encodes a number of reductases [fumarate reductase, nitrate reductase, dimethylsulfoxide (DMSO) reductase and tetrathionate reductase], which allow the bacterium to carry out respiration with several alternative electron acceptors to oxygen (fumarate, nitrate, DMSO and tetrathionate) under anaerobic conditions (Table 5). The presence of versatile reductases may give clues to the living habitats of the bacterium where alternative electron acceptors can be found in that environment. The presence of DMSO reductase and tetrathionate reductase is of particular interest. DMSO is abundant in aquatic environments which can be produced from the photochemical oxidation of dimethyl sulfide (DMS) and also from eukaryotic microplankton [46,47]. It may reflect the environment where *L. hongkongensis *is found. As for tetrathionate reductase, the presence of a complete *ttr *gene cluster suggested that *tetrathionate *can be used as an electron acceptor during anaerobic reduction of tetrathionate to thiosulfate. Notably, *L. hongkongensis *is the only bacterium that contains the *ttr *operon among the family *Neisseriaceae *members with complete genome sequences available (LHK_01476-LHK_01478). Unlike the genes for carbohydrate, lipid and amino acid metabolism and those of the respiratory chain which are phylogenetically most related to other members of the *Neisseriaceae*, these three genes are most similar to their homologues in *Thiobacillus denitrificans *ATCC 25259 and *Aromatoleum aromaticum *EbN1 which were both β-Proteobacteria (Figure 3, 4 and 5) [48,49]. The ability to respire tetrathionate is the characteristics of certain genera of *Enterobacteriaceae *including *Salmonella*, *Citrobacter*, *Yersinia *and *Proteus *[50]. It was recently reported that tetrathionate respiration provided growth benefit to *S*. Typhimurium in the lumen of inflamed intestine over other commensals/microbiota inhabited in the gut [51]. Previously, only the source of thiosulfate, but not tetrathionate, was known in mammalian hosts. Toxic hydrogen sulphide gas produced by colonic bacteria in the intestine is detoxified by colonic muscosa into thiosulfates, thus a large quantity is present in the intestinal lumen [52,53]. It is now established that thiosulfate can be converted to tetrathionate upon reaction with reactive oxygen species that are generated during inflammation [51]. The presence of complete *ttr *gene cluster in *L. hongkongensis *genome suggests that it may confer survival advantage in mammalian host in association with diarrhea. On the other hand, the source of tetrathionate in nature is not clear, but it seems likely to occur in bacterial communities that include sulfate-reducing bacteria as it has been detected in humid soils that support growth of such bacteria [50].

### Sulfur metabolism

**Figure 6:**
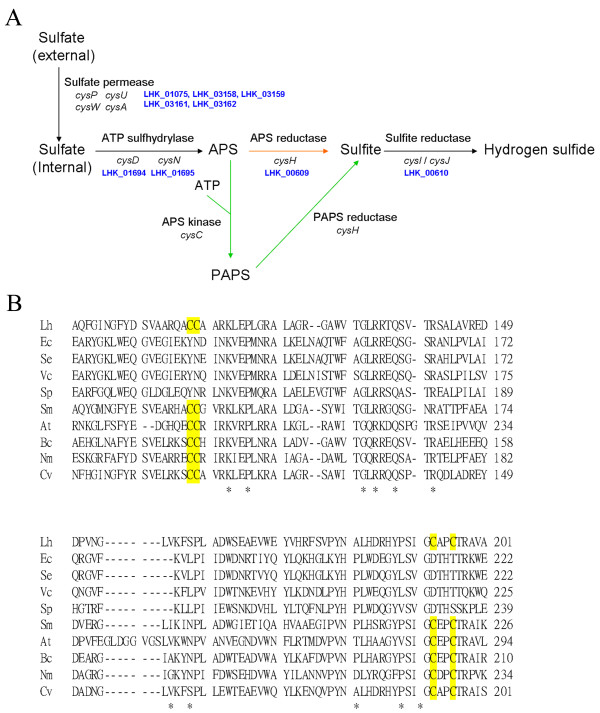
**Sulfate assimilation in *L. hongkongensis *HLHK9**. (A) Sulfate assimilation pathways in bacteria. Two pathways exist in bacteria which differ in the form of sulfate being activated. In one pathway that was once assumed to be the only one, APS is converted to PAPS by APS kinase and subsequently reduced to sulfite by PAPS reductase (green arrow). In the other pathway, APS is directly reduced to sulfite by APS reductase (orange arrow). Homologues of genes found in *L. hongkongensis *HLHK9 were specified with gene numbers in blue. APS, adenosine 5'-adenylylsulfate; PAPS, 3'-phosphoadenylylsulfate. (B) Multiple sequence alignment of predicted APS reductase in *L. hongkongensis *with other characterized or related bacterial APS and PAPS reductases. Characterized APS reductases include those from *Burkholderia cepacia*, *Arabidopsis thaliana*, *Rhizobium meliloti *whereas characterized PAPS reductases include the one from *E. coli. Only the selected region *which contains two-cysteine motifs, CCXXRKXXPL and SXGCXXCT, in the C-terminal of APS reductase is shown. The conserved cysteine residues are shaded in yellow. The sequences were aligned with ClustalW. The abbreviations used and accession numbers (shown in parentheses) are as follows: At, *A. thaliana *(GenBank:NP_193930); Bc, *B. cenocepacia *J2315 [GenBank:YP_002231786]; Cv, *C. violaceum *ATCC 12472 [GenBank:NP_903244]; Ec, *E. coli *K-12 MG1655, [GenBank:NP_417242]; Lh, *L. hongkongensis *HLHK9 [GenBank:YP_002794611]; Nm, *N. meningitidis *MC58, [GenBank:NP_274183]; Se, *Salmonella enterica *subsp. *enterica *serovar Newport str. SL317 [GenBank:ZP_02697826]; Sp, *Shewenella putrefaciens *200 [GenBank:ZP_01705880]; Sm, *Rhizobium *(*Sinorhizobium meliloti *1021) [GenBank:NP_385050] and Vc, *Vibrio cholera *TM 11079-80 [GenBank:ZP_04409338].

Sulfur is a constituent of amino acids cysteine and methionine, and also found in various coenzymes and other metabolites. The major form of inorganic sulfur in nature, sulfate, is usually incorporated into bacteria as organic compound through the pathway of sulfate assimilation. With the ability to synthesize L-cysteine and L-methionine, *L. hongkongensis *is expected to undergo sulfate assimilation with reduction of inorganic sulfate to sulfide and enter L-cysteine and L-methionine biosynthetic pathways. There are two pathways for intracellular sulfate to be reduced to sulfite and further to sulfide in bacteria, which differ in the form of activated sulfate as input (Figure 6A). The first pathway, commonly but not exclusively found in enteric bacteria such as *E. coli *and traditionally assumed to be used by the majority of bacteria, converts adenosine 5'-adenylylsulfate (APS) to 3'-phosphoadenylylsulfate (PAPS) via adenylylsulfate kinase CysC, where PAPS is eventually reduced to sulfite by PAPS reductase [54]. On the other hand, the second pathway which was originally identified in plants and subsequently found in other bacterial taxa including *P. aeruginosa*, other members of α-, β- and γ-Proteobacteria and *M. tuberculosis*, reduces APS to sulfite directly by APS reductase [55,56]. Bacterial PAPS reductase and APS reductase are highly homologous and the major difference is the presence of conserved two-cysteine motifs in APS reductase [55-57]. Phylogenetic analysis showed that APS reductase from plant and many bacteria such as *Pseudomonas*, *Rhizobium *(*Sinorhizobium*) and *M. tuberculosis *clustered together [55]. The enzyme is also assumed to be typical in β-Proteobacteria as phylogenetic analysis of the gene components suggests that homologues of APS reductase with the characteristic two-cysteine motifs are prevalently found in this group of bacteria [55]. The characteristic two-cysteine motifs, CC*XX*RK*XX*PL and S*X*GC*XX*CT, are found in the C termini of all APS reductases, but not in PAPS reductases [56]. The two-cysteine residues are reported to be involved for the binding to the iron-sulfur cluster in plant, *P. aeruginosa *and *M. tuberculosis*, which contribute to the difference in substrate specificity [58,59]. However, as this pathway has not been well characterized among β-Proteobacteria, the role of APS reductase in sulfate assimilation is uncertain in these bacterial taxa. As PAPS reductase has absolute substrate requirement, the product of the APS kinase, PAPS, is essential for *E. coli *to assimilate sulfate [54]. While the genomes of *C. violaceum *and *E. coli *are predicted to contain homologues of sulfate adenylyltransferase, APS kinase, PAPS reductase and sulfite reductase, which can encode for a complete sulfate assimilation pathway via PAPS, *L. hongkongensis *and *N. meningitidis *seem to utilize the other pathway. Sequence analysis revealed that *L. hongkongensis *and *N. meningitidis *contain homologues of APS reductase with the characteristic two-cysteine motifs, and the absence of APS kinase homologue in the genomes suggested that the reduction of sulfate to sulfite in these bacteria may not involve PAPS intermediates (Figure 6B and Table 6). Although the predicted PAPS reductase in *C. violaceum *also has the characteristic two-cysteine motifs of APS reductase (Figure 6B), it remains uncertain whether this protein catalyzes PAPS or APS.

**Table 6:** Comparison of sulfur metabolism deduced from the genomes of *L. hongkongensis*, *N. meningitidis*, *N. gonorrhoeae*, *C. violaceum*, *E. coli* and *C. jejuni*

Pathway/enzyme	Gene(s)	*L. hongkongensis *HLHK9	*C. violaceum *ATCC 12472	*N. gonorrhoeae* FA 1090	*N. meningitidis *MC58	*E. coli *K12 MG1655	*C. jejuni *NCTC 11168
Sulfate assimilation							
sulfate adenylyltransferase	*cysD, cysN*	+	+	-	+	+	+
APS kinase	*cysC*	-	+	-	-	+	+
PAPS reductase	*cysH*^a^	-	?*^b^*	-	-	+	-
APS reductase	*cysH*^a^	+	?*^b^*	-	+	-	-
Sulfite reductase	*cysI, cysJ*	+	+	+	+	+	-
Sulfate transport							
ATP binding protein CysA	*cysA*	+	+	+	+	+	-
Permease protein CysU	*cysU*	+	+	+	+	+	-
Permease protein CysW	*cysW*	+	+	+	+	+	-
Sulfate binding protein CysP	cysP	+	+	+	+	+	-

^a^Bacterial PAPS reductase and APS reductase are highly homologous and both of their gene designation are *cysH *which may represent a PAPS reductase or APS reductase [58].

^b^Predicted amino acid sequences encoded by *cysH *(CV_3574) in *C. violaceum *has the characteristic two-cysteine motifs of APS reductase and the presence of *cysC *suggests an ambiguity (represent by "?") in the identity of CV_3574 as PAPS or APS reductase.

### Nitrogen metabolism

Nitrogen is an essential component in organisms that constitutes vital biomolecules such as protein and nucleic acids. Bacteria can utilize different kinds of nitrogen compounds, including inorganic compounds such as ammonia, urea and nitrate; and organic complex compounds such as amino acids and nucleosides, as sources of cellular nitrogen. There are two general pathways found widely in bacteria for ammonia assimilation. After transport across the cytoplasmic membrane into the bacterial cell, ammonia can be incorporated into glutamate or glutamine by glutamate dehydrogenase (GDH) or glutamine synthetase/glutamate synthase (GS/GOGAT) pathway [32]. The two pathways differ according to the level of nitrogen present in the environment. When nitrogen concentration is high, ammonia assimilation via GDH is preferred as no ATP is consumed when glutamate is produced from oxoglutarate and ammonium. However, when nitrogen concentration is low, which is the case in most natural environment, ammonia is assimilated through GS/GOGAT pathway. L-glutamine is produced from L-glutamic acid and ammonia by GS in utilizing ATP and two molecules of L-glutamic acid are subsequently synthesized from L-glutamine and oxoglutarate by GOGAT because GDH has a lower affinity for ammonia. Similar to *E. coli*, *L. hongkongensis *has genes that participated in GDH and GS/GOGAT pathways, including *gdhA *that encodes NADP-dependent glutamate dehydrogenase, *gltB*/*gltD *that encode glutamate synthase and *glnA *that encodes glutamine synthetase (Table 7). On the other hand, the genomes of *C. violaceum *and *C. jejuni *contain only genes in GS/GOGAT pathway whereas those of *N. meningitidis *and *N. gonorrhoeae *contain solely genes of GS pathway. The ability of *L. hongkongensis *to utilize nitrogen is quite diverse. The existence of both pathways in *L. hongkongnesis *suggests an importance of ammonia metabolism in the living environments that it may encounter. Ammonia can be found in natural environment such as natural water and being the form of excretory waste in fish, where *L. hongkongensis *had been reported to be isolated from [6,10,60].

**Table 7:** Comparison of nitrogen metabolism deduced from the genomes of *L. hongkongensis*, *N. meningitidis*, *N. gonorrhoeae*, *C. violaceum*, *E. coli* and *C. jejuni*

Enzyme	Gene(s)	*L. hongkongensis *HLHK9	*C. violaceum* ATCC 12472	*N. gonorrhoeae* FA 1090	*N. meningitidis *MC58	*E. coli *K12 MG 1655	*C. jejuni *NCTC 11168
Glutamate dehydrogenase	*gdhA*	+	-	+	+	+	-
Glutamate synthase	*gltB/gltD*	+	+	-	-	+	+
Glutamine synthetase	*glnA*	+	+	+	+	+	+
Nitrate reductase							
Membrane-bound Nar type	*narGHJI*^a^	-	+	-	-	+	-
Periplamsic Nap type	*napABCD*^b^	+	*napA* only	-	-	+	+
Nitrite reductase	*nrfA/nifB/* *aniA/nirK*	-	+	+	+	+	-
Nitric oxide reductase	*norB*	-	+	+	+	-	-
Urease	*ureABC*^c^	+	-	-	-	-	-

^a^The basic gene components for membrane-bound type nitrate reductase to be regarded as functional [68]

^b^The basic gene components for periplasmic type nitrate reductase to be regarded as functional [68]

^c^The basic gene components for urease to be regarded as functional [77]

In addition to its presence in the natural environment, ammonia can also be provided by alternative nitrogen sources such as urea. Urea can be hydrolyzed to ammonia and carbon dioxide by urease, in which a complete urease gene cluster is found in *L. hongkongensis *and none in *N. meningitidis*, *N. gonorrhoeae*, *C. violaceum, E. coli *and *C. jejuni *(Table 7). Ammonia formed in this process not only can provide nutrient nitrogen, but may also help *L. hongkongensis *to resist acid shock during its transit through the highly acidic environment of stomach by raising pH. With alternative way to metabolize different nitrogen source such as from urea, *L. hongkongensis *can gain some survival advantages under nitrogen-limited conditions. The dual role in acid resistance and nitrogen metabolism of urease was evident in bacteria such as *Streptococcus salivarius*, *Helicboacter pylori *and *Yersinia enterocolitica *[61-63]. Investigation into the roles of urease in *L. hongkongensis *can provide insights towards the interplay of both functions and understanding about its survival strategy. Urea can be found in various environments in which *L. hongkongensis *can be found, including natural water and human host, where urea is present in a range of concentration in different parts ranging from saliva, stomach, blood to urine [64-67].

Alternatively, nitrate is another common form of nitrogen compounds found in the environment. By reducing nitrate, the majority of bacteria can incorporate nitrogen into building blocks, produce energy for cellular processes or dissipate excess energy by respiration [68]. There are three types of bacterial nitrate reductases which are classified according to their localizations and functions, namely the assimilatory (Nas type), membrane-bound (Nar-type) and periplasmic (Nap-type), of which the Nas type is used for incorporating nitrate into building blocks [68,69]. In the assimilatory pathway, nitrate is first converted to nitrite by assimilatory nitrate reductase and then to ammonium by nitrite reductase such that it can be incorporated into cell materials. Absence of the genes that encode Nas type nitrate reductase and nitrite reductase in *L. hongkongensis *genome suggested that no assimilatory pathway is present. For the respiratory pathway which mainly involved Nar and Nap type, while *E. coli *contains both types of nitrate reductases, *L. hongkongensis *and *C. jejuni *contain only periplasmic nitrate reductase whereas *C. violaceum *contains the membrane-bound type (Table 7). Even though *C. violaceum *contains *napA *that belongs to Nap system, the absence of other gene components suggested that this pathway is incomplete and probably non-functional. On the other hand, no homologues of nitrate reductases are found in *N. meningitidis *and *N. gonorrhoeae*. The membrane-bound nitrate reductase, encoded by NarGHI operon, can be found in many nitrate-respiring and denitrifying bacteria and functions as the generator of proton motive force by coupling nitrate reduction in respiration. In contrast to Nar, the function of Nap is much diverse and appears to differ among bacteria. Apart from its involvement in anaerobic respiration in bacteria such as *E. coli*, it can have other physiological roles such as participating in redox balancing for optimal bacterial growth under certain physiological conditions and aerobic denitrification switch from aerobic respiration to denitrification or scavenges nitrate in some pathogenic bacteria [68]. The gene composition and ordering of *nap *gene cluster shows heterogenicity amongst different bacteria, with *napD *and *napA *as the only genes that are always found in the cluster [68]. In *L. hongkongensis*, it is predicted that *nap *operon contains 7 genes, *napFDAGHBC*, with the same gene composition and order as in *E. coli*. The ability for nitrate reduction in *L. hongkongensis *suggested that Nap type nitrate reductase is functional [1,3,11]. Apart from the above pathways, an absence of nitric oxide reductase in *L. hongkongensis *suggested that dissimilatory pathway for the production of dinitrogen is not operational.

## Conclusions

The *L. hongkongensis *genome possesses a variety of genes and pathways for carbohydrate, amino acid and lipid metabolism, respiratory chain and sulfur and nitrogen metabolism. These allow the bacterium to utilize various substrates for energy production and survive in different environmental niches.

## Methods

CDSs identified in the *L. hongkongensis *genome were annotated as described in our previous publication and classified functionally according to the COG methodology [11]. Annotated genes were mapped to pathways according to the Kyoto Encyclopedia of Genes and Genomes (KEGG) database and MetaCyc to help identify metabolic pathways, and refined with thorough literature mining and experimental data [70,71]. When "gap" exists in the metabolic pathways, reciprocal-best-hit search was adopted to identify orthologues using experimentally verified protein sequence as query. CDSs belonging to metabolism-related COG clusters (C, G, E, I, and P) were selected for further examination and review. Other CDSs of potential interest were identified by keyword search using the names of common amino acids, carbohydrates, nucleotides, coenzymes, lipids, inorganic ions and metabolites. Manual annotation and analysis of the assigned function was performed by sequence similarity search using BLAST against the NCBI nr database, and assisted by conserved domain search (CD-search), identification of signature sequence motifs and sequence analysis using InterProScan. Localization patterns were predicted using PSORTb where appropriate [72]. Comparison of CDSs belonging to metabolism-related COG clusters among *L. hongkongensis *HLHK9, *C. violaceum *ATCC 12472, *N. meningitidis *MC58, *N. gonorrhoeae *FA 1090, *E. coli *K12 MG1655 and *C. jejuni *NCTC 11168 was performed using KEGG database, MetaCyc and Integrated Microbial Genomes (IMG) database [73]. Phylogenetic trees were constructed by the neighbor-joining method with MEGA 4.0 using Kimura's two-parameter correction for 16S rRNA gene and Poisson correction for tetrathionate reductase [74]. 1553 nucleotide positions of 16S rRNA gene, and 1026, 233 and 389 amino acid positions of *ttrA*, *ttrB *and *ttrC *genes encoded proteins respectively were included in the analysis.

## List of abbreviations

Acetyl-CoA: acetyl-coenzyme A; ACP: acyl-carrier protein; Acyl-CoA: acyl-coenzyme A; ATP: adenosine triphosphate; APS: adenosine 5'-adenylylsulfate; CDS(s): coding sequence(s); CFA: cyclopropane fatty-acyl-phospholipid; COG: cluster of Orthologous Group; DMS: dimethyl sulfide; DMSO: dimethylsulfoxide; DNA: deoxyribonucleic acid; ED: Entner-Doudoroff; EI: enzyme I; EMP: Embden-Meyerhof-Parnas; FAD: flavin adenine dinucleotide; FabA: 3-hydroxydecanoyl-(acyl-carrier-protein)-dehydratase; FabB: hydroxylacyl-(acyl-carrier-protein) synthase I; FabM: *trans-2: cis-3*-decenoyl-ACP isomerase; FabZ: beta-hydroxyacyl-(acyl-carrier-protein) dehydratase; FabF: beta-ketoacyl-(acyl carrier protein) synthase II; GDH: glutamate dehydrogenase; GS/GOGAT: glutamine synthetase/glutamate synthase; HPr: phosphocarrier protein; HPrK: phosphocarrier protein kinase/phosphorlyase; KEGG: Kyoto Encyclopedia of Genes and Genomes; NADH: nicotiamide adenine dinucleotide; NAGK: *N*-acetyl-L-glutamate kinase; NAGK-20: *N-*acetyl-L-glutamate kinase with higher expression at 20°C; NAGK-37: *N-*acetyl-L-glutamate kinase with higher expression at 37°C; Nas: assimilatory nitrate reductase; Nar: membrane-bound nitrate reductase; Nap: periplasmic nitrate reductase; NDH: NADH dehydrogenase; NPr: variant of phosphocarrier protein; PAPS: 3'-phosphoadenylylsulfate; PPP: pentose phosphate pathway; PTS: phosphotransferase system; TCA: tricarboxylic acid; *Sec: selenocysteine; *UFA(s): unsaturated fatty acid(s)

## Competing interests

The authors declare that they have no competing interests.

## Authors' contributions

PCW, KYY and SKL designed and supervised the study. SOC and JLT annotated the genome. HT performed bioinformatics analysis. SOC and PCW drafted the manuscript. All authors read, corrected and approved the final manuscript.
